# Mindfulness and psychological distress among Chinese taekwondo athletes: the mediating roles of stress, self-management, and brooding

**DOI:** 10.3389/fpsyg.2026.1904502

**Published:** 2026-07-31

**Authors:** Binbin Liu, Hangyu Peng, Qiwei Yang

**Affiliations:** 1 Department of Physical Education, Hoseo University, Asan-si, Republic of Korea; 2 School of Physical Education, Hunan University of Science and Technology, Xiangtan, China

**Keywords:** brooding, mindfulness, psychological distress, stress self-management, taekwondo athletes

## Abstract

**Introduction:**

Taekwondo athletes are frequently exposed to intensive training demands, competitive pressure, and injury-related stress, all of which may increase their vulnerability to psychological distress. Although mindfulness has been widely recognized as a protective psychological resource, less is known about how it is associated with psychological distress among taekwondo athletes, particularly through self-regulatory and cognitive mechanisms. Therefore, this study examined the association of mindfulness with psychological distress among taekwondo athletes and further explored the mediating roles of stress self-management and brooding.

**Methods:**

A cross-sectional survey was conducted in March 2026 among 346 taekwondo athletes recruited using a convenience sampling approach from university teams, sports schools/professional training teams, and competitive clubs in China. Participants completed measures of mindfulness, stress self-management, brooding, and psychological distress. Data were analyzed using IBM SPSS 26.0 and AMOS 26.0. Structural equation modeling was employed to test the hypothesized relationships, and bootstrapping was used to examine the indirect effect.

**Results:**

The structural model showed a good fit to the data. After controlling for training frequency and sports injury experience, mindfulness was positively associated with stress self-management (β = 0.435, *p* < 0.001) and negatively associated with brooding (β = −0.258, *p* < 0.001). Stress self-management was negatively associated with both brooding (β = −0.351, *p* < 0.001) and psychological distress (β = −0.290, *p* < 0.001), whereas brooding was positively associated with psychological distress (β = 0.335, *p* < 0.001). The standardized indirect effect of mindfulness on psychological distress through stress self-management and brooding was significant (β = −0.264, SE = 0.040, 95% CI [−0.342, −0.187], *p* < 0.001). The model explained 19.3% of the variance in stress self-management, 26.9% of the variance in brooding, and 28.7% of the variance in psychological distress.

**Discussion:**

Mindfulness was associated with lower psychological distress among taekwondo athletes, both directly and indirectly through higher stress self-management and lower brooding. These findings highlight the importance of mindfulness as a psychological resource in athletic settings and suggest that interventions aimed at improving stress self-management and reducing maladaptive rumination may help promote athletes' mental wellbeing.

## 1 Introduction

Elite athletes are routinely exposed to intensive physical demands, rigorous training schedules, and highly competitive environments, all of which may impose substantial psychological pressure. Consequently, psychological distress has become an increasingly important mental health concern within athletic populations. While early landmark studies historically conceptualized athletes as resilient cohorts relatively immune to psychiatric vulnerabilities (Hughes and Coakley, 1991), substantial classical literature has since established that competitive athletes suffer from mental health disorders at rates comparable to, or even exceeding, the general population (Rice et al., 2016). Existing evidence indicates that approximately 19.6% of elite athletes report symptoms of psychological distress, while up to 34% experience anxiety and depressive symptoms (Gouttebarge et al., 2019). In addition, more than half of elite athletes have reportedly encountered mental health problems at some point in their lives, with a considerable proportion reaching clinically significant levels (Akesdotter et al., 2020). Longitudinal evidence further suggests that psychological distress among athletes is not merely temporary but may persist over time, with more than 40% of athletes experiencing symptoms of anxiety or depression during extended follow-up periods (Bentzen et al., 2025). Such findings highlight that psychological distress is both prevalent and persistent in competitive sports and may adversely affect athletes' wellbeing, performance, and long-term career development.

Among various athletic populations, taekwondo athletes may be particularly vulnerable to psychological distress due to the distinctive demands associated with combat sports. Taekwondo is characterized by intensive physical confrontation, rapid decision-making under competitive pressure, and high-intensity training regimens, all of which increase athletes' physical and psychological burden (Huang et al., 2025). Previous research has shown that taekwondo athletes are exposed to elevated risks of both acute and chronic injuries because of repetitive high-impact movements and intensive competition demands (Willauschus et al., 2021). Moreover, the weight-class system in taekwondo often requires athletes to engage in strict body weight management and rapid weight-loss practices, which may further intensify physiological strain and psychological stress (Andreato et al., 2022; Casolino et al., 2012). Existing evidence also suggests that athletes' performance and injury risk are influenced not only by physiological capacity but also by psychological readiness, reflecting the close interconnection between physical and psychological functioning in taekwondo (Aldhahi et al., 2026). Taken together, these sport-specific characteristics suggest that taekwondo athletes represent a population facing considerable psychological challenges, thereby underscoring the importance of examining factors associated with their mental health outcomes.

In response to the growing concern regarding athletes' mental health, mindfulness has attracted increasing scholarly attention as a potentially beneficial psychological resource. Rooted in classical Buddhist traditions and popularized in contemporary psychology by Kabat-Zinn et al. (1986), mindfulness is widely defined as the awareness that emerges through paying attention on purpose, in the present moment, and nonjudgmentally to the unfolding of experience. Mindfulness is generally conceptualized as receptive attention to and awareness of present-moment experiences (Brown and Ryan, 2003), and has its origins in the work of (Barker, 2014), who described mindfulness as purposeful, present-centered, and nonjudgmental awareness. A substantial body of research has demonstrated that mindfulness is associated with enhanced psychological wellbeing and lower levels of anxiety, depression, and emotional distress across both general and clinical populations (Bos et al., 2024; Guendelman et al., 2017; Hofmann and Gómez, 2017; Paz and Davidovitch, 2025; Keng et al., 2011).

Within sport settings, emerging evidence likewise suggests that mindfulness may be relevant to athletes' mental health and performance-related outcomes (Rogowska and Tataruch, 2024; Wang et al., 2023). This line of research aligns with established sports psychology frameworks, notably the Mindfulness-Acceptance-Commitment approach developed by Gardner and Moore (2012), which suggests that non-judgmental acceptance of internal experiences reduces performance-related distress. For example, a recent systematic review and meta-analysis reported that mindfulness-based programs significantly improved mental health outcomes among elite athletes, including reductions in anxiety and stress as well as improvements in psychological wellbeing (Myall et al., 2023). Despite these promising findings, research examining mindfulness in relation to psychological distress among taekwondo athletes remains relatively limited.

Although prior studies have increasingly linked mindfulness to athletes' mental health outcomes, growing attention has also been devoted to understanding the psychological processes associated with these relationships. In competitive sports, athletes are required not only to cope with intensive training and performance-related stressors but also to continuously regulate their emotional, behavioral, and cognitive responses under pressure. Accordingly, recent sport psychology research has increasingly emphasized the role of self-regulatory and cognitive processes in athletes' psychological adaptation and wellbeing (Latinjak, 2025; Hufton et al., 2024), aligning with foundational theories that position self-regulation as a core determinant of athletic expertise and emotional control (Jonker et al., 2010; Toering et al., 2009). From the perspective of self-regulation theory, these mechanisms can be explained through individuals' continuous processes of self-monitoring and adjustment to maintain adaptive functioning. Specifically, stress self-management functions as an active behavioral regulatory process that allows athletes to maintain psychological equilibrium when environmental demands outweigh personal resources (Yu and Pei, 2026). Conversely, brooding acts as a maladaptive cognitive regulatory tendency characterized by a passive, repetitive focus on distress symptoms, which disrupts effective problem-solving (Deliu et al., 2026; Roy et al., 2016; Blankstein and Lumley, 2008). By applying a self-regulation framework, it becomes clear that understanding athletes' psychological distress requires explaining both the behavioral strategies used to manage external stressors and the cognitive mechanisms that process internal negative experiences.

Despite the growing body of research on mindfulness and athletes' mental health, several important areas warrant further investigation. First, existing research has predominantly focused on athletes from general sport populations (Cui et al., 2025; Yiyi et al., 2025), whereas relatively limited attention has been devoted to taekwondo athletes. Given the distinctive characteristics of taekwondo, including intensive physical confrontation, weight-management pressure, and elevated injury risk, further research within this specific combat sport context may broaden current understanding of athletes' mental health and provide evidence from a population facing unique psychological demands. Second, previous studies have demonstrated that both self-regulatory factors and cognitive factors are important for athletes' psychological functioning (Scharfen and Memmert, 2019; García et al., 2023). However, these two lines of research have largely developed independently. In particular, stress self-management, which reflects athletes' adaptive self-regulatory capacity, and brooding, which represents a maladaptive cognitive tendency, have seldom been examined simultaneously when investigating psychological distress among athletes. Considering these processes within the same framework may therefore provide a more comprehensive understanding of athletes' psychological adaptation. Third, although previous research has established that mindfulness is associated with a range of positive mental health outcomes in sport settings, relatively limited attention has been devoted to explaining how mindfulness may be associated with psychological distress from a theoretical perspective. In particular, the psychological processes linking mindfulness to psychological distress have not been sufficiently examined within the framework of self-regulation theory. Applying this theoretical perspective may contribute to a deeper understanding of how adaptive self-regulatory processes and maladaptive cognitive processes jointly shape psychological distress among taekwondo athletes.

Accordingly, the present study aims to examine the association between mindfulness and psychological distress among taekwondo athletes, with particular attention to the mediating roles of stress self-management and brooding. This study contributes to the existing literature in several ways. First, by focusing specifically on taekwondo athletes, the present study extends existing research on mindfulness and athletes' mental health to a combat sport context characterized by intensive physical demands, weight-management pressure, and elevated injury risk. Second, the present study contributes to the sport psychology literature by examining stress self-management and brooding concurrently when investigating psychological distress among athletes, thereby broadening current understanding of the self-regulatory and cognitive processes associated with athletes' mental health. Third, by drawing upon self-regulation theory, this study provides additional insight into the psychological mechanisms through which mindfulness may be associated with psychological distress in competitive sport settings. Fourth, the present study may also provide practical implications for athlete mental health management by offering a better understanding of the psychological factors associated with psychological distress among taekwondo athletes, which may help inform the development of mindfulness-based interventions and psychological support strategies in athletic settings.

The remainder of this study is organized as follows. Section 2 reviews the relevant literature, presents the theoretical foundation, and develops the research hypotheses. Section 3 describes the research methodology, including the sample, measurement instruments, and data analysis procedures. Section 4 reports the empirical results and examines the proposed relationships among the study variables. Section 5 discusses the main findings, theoretical contributions, practical implications, and study limitations, and provides directions for future research. Finally, Section 6 concludes the study.

## 2 Literature review and hypothesis development

### 2.1 Self-regulation theory

Self-regulation theory conceptualizes human behavior as a continuous process in which individuals monitor their internal states and external environments, evaluate discrepancies between current and desired states, and adjust their thoughts, emotions, and behaviors accordingly in order to achieve adaptive functioning (Carver and Scheier, 1982; Bandura, 1991). From this perspective, psychological adaptation depends on the effective operation of regulatory processes that enable individuals to manage stressors and maintain goal-directed behavior in dynamic and demanding contexts. Failures or inefficiencies in self-regulation are considered key mechanisms underlying maladaptive emotional and cognitive responses, which may contribute to psychological distress (Baumeister and Heatherton, 1996).

Within this theoretical framework, self-regulation is not limited to behavioral control but also encompasses emotional and cognitive regulatory processes. In sport settings, behavioral self-regulation is reflected in athletes' capacity to manage training demands, competitive pressure, and stress-related experiences in a goal-directed manner, which is commonly conceptualized as stress self-management Van Dam et al., (2010; Nicholls and Polman, 2007). In contrast, cognitive dysregulation may manifest in maladaptive repetitive thinking patterns that interfere with effective adaptation. Brooding, as a form of persistent negative self-focused cognition, represents a maladaptive cognitive regulatory process that reflects difficulties in disengaging from negative experiences and has been consistently associated with impaired psychological functioning (Treynor et al., 2003; Nolen-Hoeksema et al., 2008). Accordingly, both stress self-management and brooding can be understood as distinct but theoretically connected manifestations of self-regulatory processes in achievement-oriented contexts.

Building on self-regulation theory, mindfulness can be conceptualized as a psychological resource that facilitates effective self-regulatory functioning. Rather than operating as an outcome of regulation, mindfulness reflects an attentional and awareness-based capacity that enables individuals to observe present-moment experiences with greater clarity and reduced automatic reactivity (Brown and Ryan, 2003). This enhanced awareness is considered to support more adaptive monitoring and adjustment processes, thereby strengthening behavioral self-regulation while simultaneously reducing the likelihood of engaging in maladaptive cognitive patterns. In highly demanding sport environments such as taekwondo, where athletes must continuously respond to performance pressure, physical confrontation, and evaluative stressors, mindfulness may therefore play a central role in facilitating adaptive self-regulation.

Overall, self-regulation theory provides a coherent theoretical foundation for the present study by explaining how mindfulness may be associated with psychological distress through both behavioral and cognitive regulatory pathways. Specifically, it suggests that mindfulness may enhance athletes' ability to engage in adaptive stress self-management while simultaneously reducing maladaptive cognitive tendencies such as brooding, thereby shaping overall psychological adjustment in competitive sport contexts.

### 2.2 Hypothesis development

#### 2.2.1 Mindfulness, stress self-management, and brooding

Mindfulness has been widely regarded as an important psychological resource associated with adaptive self-regulatory functioning. Existing research suggests that mindfulness is characterized by present-moment awareness and nonjudgmental attention to ongoing experiences, which may facilitate individuals' ability to regulate emotional and behavioral responses under stressful conditions (Bigman-Peer and Yovel, 2024). Previous studies have shown that individuals with higher levels of mindfulness generally demonstrate better coping ability (Keng et al., 2018), emotional regulation (Roemer et al., 2015), and stress management capacity (Sharma and Rush, 2014; Petterson and Olson, 2017). Within sport settings, mindfulness has also been linked to athletes' psychological adaptation and their ability to manage competitive pressure and performance-related stressors (Josefsson et al., 2017; Birrer et al., 2012). For example, mindfulness-based interventions have been associated with improvements in football players' emotional regulation and stress-related outcomes (Norouzi et al., 2020). Since stress self-management reflects athletes' capacity to regulate and manage stressful competitive demands, mindfulness may be relevant to more adaptive stress self-management among taekwondo athletes. Therefore, this study proposes the following hypothesis:

**Hypothesis 1 (H1):** mindfulness is positively associated with stress self-management among taekwondo athletes.

In addition to behavioral and emotional regulation, previous research has increasingly linked mindfulness to cognitive processes associated with mental health outcomes. Brooding, which represents a maladaptive form of repetitive negative thinking, has been identified as an important cognitive factor related to psychological maladjustment and emotional distress (Ren et al., 2025; Olson and Kwon, 2008). Existing evidence suggests that mindfulness is associated with lower levels of maladaptive rumination and repetitive negative thinking because mindful individuals tend to maintain greater awareness of present-moment experiences rather than becoming excessively engaged in negative self-focused thoughts (Keng et al., 2016; Schlosser et al., 2022). In athletic populations, mindfulness has also been associated with reduced cognitive interference and improved attentional regulation among athletes (Yang et al., 2025; Ding et al., 2025). This may be particularly relevant among taekwondo athletes, as the highly competitive and confrontational nature of the sport may increase athletes' vulnerability to repetitive negative thinking following performance mistakes or competitive setbacks (Bridge et al., 2014; Yu et al., 2026). In this context, mindfulness may help athletes maintain present-moment awareness and reduce excessive cognitive engagement with negative performance-related experiences. Therefore, this study proposes the following hypothesis:

**Hypothesis 2 (H2):** mindfulness is negatively associated with brooding among taekwondo athletes.

#### 2.2.2 Stress self-management, brooding, and psychological distress

Stress self-management represents a proactive and goal-directed process where individuals utilize behavioral and cognitive strategies to maintain psychological equilibrium (Hughes et al., 2006; Zhang et al., 2024). In contrast, brooding is defined as a passive and repetitive focus on the symptoms of distress and the possible causes and consequences of such distress (Blankstein and Lumley, 2008; Clancy et al., 2020). Empirical research suggests that an increase in active coping and self-regulatory skills can effectively inhibit maladaptive cognitive patterns (Ong and Thompson, 2019; Brzozowski and Philip Crossey, 2024). When athletes possess a higher capacity for stress self-management, they are more likely to transition from a state of emotional arousal to task-oriented problem-solving, thereby reducing the cognitive resources available for ruminative processes such as brooding (Nuetzel, 2025, 2023). In the specific context of taekwondo, athletes who effectively manage competitive pressures and weight-related stressors are less likely to engage in the repetitive negative thinking that characterizes brooding. Therefore, this study proposes the following hypotheses:

**Hypothesis 3 (H3):** stress self-management is negatively associated with brooding among taekwondo athletes.

The capacity to manage stress is widely recognized as a fundamental protective factor for mental health in competitive sports (Song et al., 2025). Self-regulation theory posits that psychological distress often arises when individuals perceive a significant discrepancy between environmental demands and their regulatory resources (Bakker and de Vries, 2021). Previous studies have further shown that adaptive stress management and coping strategies are associated with lower levels of anxiety, emotional exhaustion, and psychological maladjustment among athletes (Wilks, 1991; Suinn, 2005). In taekwondo, athletes face frequent physical confrontation and high-stakes performance expectations, both of which can lead to significant psychological strain. Effective stress self-management allows these athletes to mitigate the impact of these stressors by maintaining emotional stability and cognitive clarity (Kim et al., 2022). By successfully navigating the demands of rigorous training and competition through systematic self-regulation, athletes can prevent the accumulation of psychological pressure and maintain higher levels of psychological wellbeing. Therefore, this study proposes the following hypotheses:

**Hypothesis 4 (H4):** stress self-management is negatively associated with psychological distress among taekwondo athletes.

Brooding has been identified as a maladaptive form of rumination involving repetitive and passive focus on negative emotions and distress-related experiences (Orue et al., 2014; Burwell and Shirk, 2007). Previous research has consistently linked brooding to adverse mental health outcomes, including anxiety, depression, and emotional distress (Lackner and Fresco, 2016; Jose and Weir, 2013; Borg et al., 2022). Individuals with higher levels of brooding are more likely to become cognitively preoccupied with negative experiences and emotional difficulties, which may intensify psychological maladjustment over time (Watkins, 2009; Burwell, 2015). Within athletic contexts, repetitive negative thinking related to performance failure, injury concerns, or competitive pressure may similarly contribute to poorer psychological functioning among athletes. Given the highly competitive and evaluative nature of taekwondo, athletes who engage in brooding may be more vulnerable to psychological distress. Therefore, this study proposes the following hypothesis:

**Hypothesis 5 (H5):** brooding is positively associated with psychological distress among taekwondo athletes.

#### 2.2.3 Mediation effects

From the perspective of self-regulation theory, mindfulness may be associated with psychological outcomes through multiple self-regulatory and cognitive processes (Schultz and Ryan, 2015). Previous research has shown that mindfulness is related to adaptive self-regulatory functioning, including improved emotional regulation (Tang et al., 2016), coping ability (Dvoráková et al., 2019), and stress management (Yang et al., 2018). In sport settings, mindfulness has similarly been associated with athletes' psychological adaptation and their ability to manage competitive stressors more effectively (Röthlin et al., 2020; Liu and Noh, 2024). At the same time, existing evidence suggests that mindfulness is negatively related to maladaptive cognitive processes such as rumination and repetitive negative thinking (Maltais et al., 2019). These findings indicate that mindfulness may influence psychological wellbeing not only through behavioral self-regulation but also through cognitive regulatory processes.

**Figure 1:**
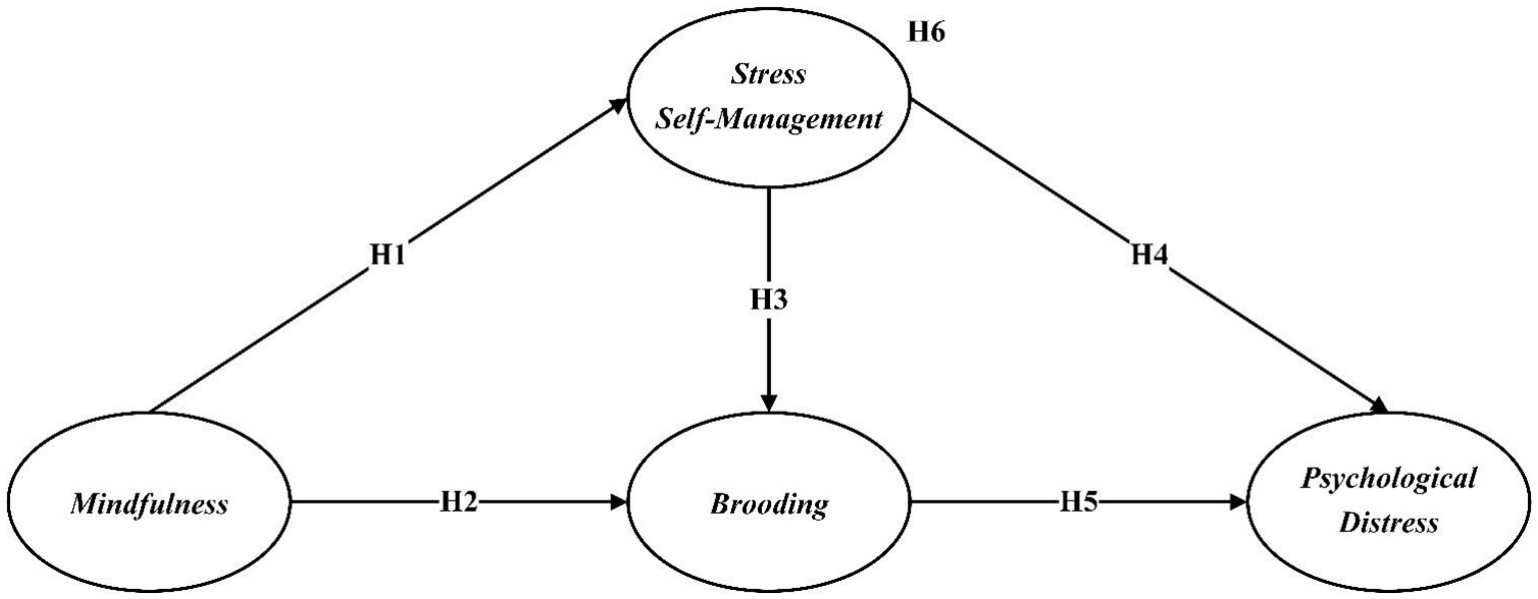
Hypothesis model.

In addition, previous studies have suggested that adaptive stress management is associated with lower levels of maladaptive cognitive responses and better mental health outcomes (Holton et al., 2016; Antoni et al., 2023), whereas brooding has been consistently linked to elevated psychological distress (Kokonyei et al., 2016). In highly demanding sport environments such as taekwondo, athletes are frequently required to regulate stress, performance-related thoughts, and emotional responses under pressure. Accordingly, stress self-management and brooding may represent two interrelated psychological processes associated with athletes' mental health adaptation. Although previous research has examined mindfulness, stress management, rumination, and psychological distress separately, relatively limited research has integrated these factors within the same framework among taekwondo athletes. Based on self-regulation theory and the existing literature, mindfulness may therefore be indirectly associated with psychological distress through stress self-management and brooding. Therefore, this study proposes the following hypothesis:

**Hypothesis 6 (H6):** mindfulness is indirectly associated with psychological distress through the mediating roles of stress self-management and brooding among taekwondo athletes. All hypotheses are summarized in Figure 1.

## 3 Methodology

### 3.1 Participants and procedures

Participants were recruited in March 2026 from taekwondo teams and training settings that were accessible to the research team, including university taekwondo teams, sports schools/professional training teams, and competitive taekwondo clubs in China. A non-probability convenience sampling approach was adopted because the study targeted athletes who were currently involved in regular taekwondo training and could be practically reached through existing institutional and coaching contacts. Therefore, the sample represented available taekwondo athletes from participating teams and training settings rather than a randomly selected national sample.

Before data collection, the researchers contacted team coaches, instructors, or relevant coordinators to explain the purpose of the study and obtain permission for questionnaire distribution. After approval was obtained, the questionnaires were administered either in person after training sessions or through organized online distribution to eligible participants in the corresponding teams or groups. Participation was voluntary, and all respondents were informed that their answers would be used only for academic research and kept confidential. Only those who agreed to participate were invited to complete the questionnaire.

To ensure the relevance of the sample, participants were required to be currently engaged in taekwondo training and to have formal taekwondo competition experience. After data screening, responses with substantial missing values, obvious response patterns, or inconsistent answers were excluded. A total of 346 valid questionnaires were retained for the final analysis.

As shown in Table 1, the sample consisted of 346 taekwondo athletes with a relatively balanced gender distribution. Most participants were young adults, with nearly half aged 22–24 years. In terms of primary performance type, almost half were from professional teams or sports schools. The participants generally reported relatively frequent training, with the largest proportion training 5–6 times per week, and most had accumulated several years of formal competition experience. In addition, a large majority reported having experienced sports injuries affecting training or competition during the past 12 months. Given the convenience sampling strategy, the findings should be interpreted with caution when extrapolating to all Chinese taekwondo athletes or to athletes in other sports and training contexts.

**Table 1:** Demographic characteristics (*n* = 346).

Variable	Category	%
Gender	Male	52.9
	Female	47.1
Age	18–21 years	30.6
	22–24 years	48.0
	25–27 years	16.5
	28 years or above	4.9
Primary performance type	University taekwondo athlete	34.1
	Professional team/sports school athlete	49.7
	Competitive club athlete	16.2
Training frequency	1–2 times/week	6.6
	3–4 times/week	32.1
	5–6 times/week	41.9
	7 or more times/week	19.4
Competition experience	2 years or less	22.3
	3–4 years	43.6
	5 years or more	34.1
Sports injury experience (past 12 months)	Yes	85.5
	No	14.5

### 3.2 Instruments

Four established instruments were used to assess mindfulness, stress self-management, brooding, and psychological distress. All items were rated on a 5-point Likert scale. Because the original instruments were developed in English, the present study used Chinese questionnaire items prepared through a standardized translation and cultural adaptation procedure. First, two bilingual researchers independently translated the English items into Chinese. Second, the translated items were compared and discussed to produce a reconciled Chinese version. Third, another bilingual researcher, who was not involved in the initial translation, back-translated the Chinese items into English. The back-translated version was then compared with the original English scales to identify and revise any discrepancies in wording or meaning. Finally, two sport psychology researchers and one taekwondo coach reviewed the Chinese items to ensure linguistic clarity, cultural appropriateness, and relevance to Chinese taekwondo athletes. Minor wording adjustments were made to improve readability without changing the conceptual meaning of the original items.

Before the formal survey, the Chinese questionnaire was pilot-tested with a small group of taekwondo athletes to assess item comprehension and response clarity. Participants reported that the items were understandable and appropriate for the athletic context. In the formal sample, the reliability and construct validity of the Chinese versions were further evaluated using Cronbach's alpha, composite reliability, average variance extracted, and confirmatory factor analysis. Because this study involved only Chinese athletes and did not compare groups across language versions or cultural groups, cross-language measurement invariance was not tested. Instead, the factorial validity and internal consistency of the Chinese instruments were examined within the present sample.

Mindfulness was measured using the 5-item Mindful Attention Awareness Scale (MAAS-5) developed by (Van Dam et al. 2010). Participants responded on a 5-point scale ranging from one (strongly disagree) to five (strongly agree). A sample item is: “It seems I am ‘running on automatic,' without much awareness of what I'm doing.” Because the mindfulness items were negatively worded, they were reverse-scored so that higher scores indicated higher levels of mindfulness.

Stress self-management was assessed using the stress management subscale of the Health-Promoting Lifestyle Profile developed by (Pinar et al., 2009). The scale consists of six items rated from one (never) to five (always). A sample item is: “Use stress control methods.” Higher scores indicated better stress self-management.

Brooding was measured using the 5-item brooding subscale of the Ruminative Responses Scale, based on Nolen-Hoeksema (1991). Participants rated each item on a 5-point scale ranging from one (never) to five (always). A sample item is: “Think ‘What am I doing to deserve this?”' Higher scores reflected higher levels of brooding.

Psychological distress was assessed using the K6 scale developed by Kessler et al. (2002). This instrument contains six items measuring non-specific psychological distress over the past 30 days. Responses were rated on a 5-point scale ranging from one (never) to five (always). A sample item is: “During the last 30 days, about how often did you feel so depressed that nothing could cheer you up?” Higher scores indicated greater psychological distress.

### 3.3 Data analysis

All data were analyzed using IBM SPSS 26.0 and AMOS 26.0. SPSS 26.0 was used for data screening, descriptive statistics, reliability analysis, and Pearson correlation analysis. AMOS 26.0 was used to conduct confirmatory factor analysis (CFA) and structural equation modeling (SEM) to examine the hypothesized relationships among mindfulness, stress self-management, brooding, and psychological distress. The measurement model was first assessed in terms of reliability and validity, followed by testing of the structural model.

Training frequency and sports injury experience during the past 12 months were included as control variables in the structural model. Training frequency was treated as an ordinal variable, with higher values indicating more frequent weekly training, whereas sports injury experience was coded as 0 = no and 1 = yes. Both control variables were specified as predictors of stress self-management, brooding, and psychological distress. Mindfulness, training frequency, and sports injury experience were treated as exogenous variables and were allowed to covary. Finally, bootstrapping with 5,000 resamples and bias-corrected 95% confidence intervals was performed to examine the significance of the indirect effect.

To assess the potential influence of common method bias, Harman's single-factor test was conducted. The results showed that the first unrotated factor accounted for 35.089% of the total variance, which was below the commonly accepted threshold of 40%. In addition, because Harman's single-factor test alone may be insufficient, a common latent factor approach was conducted in AMOS to further assess common method variance. The results indicated that adding a common latent factor did not substantially change the standardized factor loadings or the interpretation of the measurement model. Therefore, common method bias was unlikely to seriously affect the main findings of this study.

## 4 Results

### 4.1 Measurement model

To assess the adequacy of the measurement model, a confirmatory factor analysis was performed for the four latent constructs: mindfulness, stress self-management, brooding, and psychological distress. The results showed that all item loadings were statistically acceptable, ranging from 0.720 to 0.802, indicating that the indicators adequately represented their respective latent variables.

As reported in Table 2, Cronbach's alpha values ranged from 0.859 to 0.895, while composite reliability values ranged from 0.860 to 0.895, both exceeding the recommended threshold of 0.70. Furthermore, the average variance extracted values ranged from 0.551 to 0.588, all above 0.50. These findings support the internal consistency and convergent validity of the measurement model.

**Table 2:** Results of the measurement model.

Items	Loadings	Cronbach's α	CR	AVE
Mindfulness (MIN)		0.859	0.860	0.551
MI1	0.753			
MI2	0.746			
MI3	0.771			
MI4	0.720			
MI5	0.720			
Stress self-management (SSM)		0.895	0.895	0.588
SS1	0.736			
SS2	0.740			
SS3	0.791			
SS4	0.753			
SS5	0.802			
SS6	0.777			
Brooding (BR)		0.869	0.869	0.571
BR1	0.730			
BR2	0.743			
BR3	0.791			
BR4	0.741			
BR5	0.770			
Psychological distress (PD)		0.890	0.890	0.574
PD1	0.737			
PD2	0.799			
PD3	0.776			
PD4	0.746			
PD5	0.750			
PD6	0.735			

Discriminant validity was evaluated based on the Fornell–Larcker criterion. Table 3 shows that the square root of the AVE for each construct was greater than its correlations with the other constructs. Specifically, the square roots of the AVEs were 0.742 for mindfulness, 0.767 for stress self-management, 0.756 for brooding, and 0.758 for psychological distress. Therefore, discriminant validity was supported.

**Table 3:** Correlations and discriminant validity.

Construct	MI	SS	BR	PD
MI	(0.742)			
SS	0.376^**^	(0.767)		
BR	−0.343^**^	−0.408^**^	(0.756)	
PD	−0.360^**^	−0.392^**^	0.409^**^	(0.758)

Values on the diagonal represent the square root of AVE, while off-diagonals indicate Pearson correlations between constructs. ^**^ indicates that the correlation is statistically significant at *p* < 0.01.

### 4.2 Structural model

After confirming the adequacy of the measurement model, the structural model was tested with training frequency and sports injury experience during the past 12 months included as control variables. The results indicated that the structural model fit the data well, with χ^2^ = 278.708, df = 240, χ^2^/df = 1.161, RMR = 0.049, GFI = 0.936, AGFI = 0.920, IFI = 0.990, TLI = 0.988, CFI = 0.990, and RMSEA = 0.022. Overall, these fit indices suggested that the structural model had a good fit.

**Figure 2:**
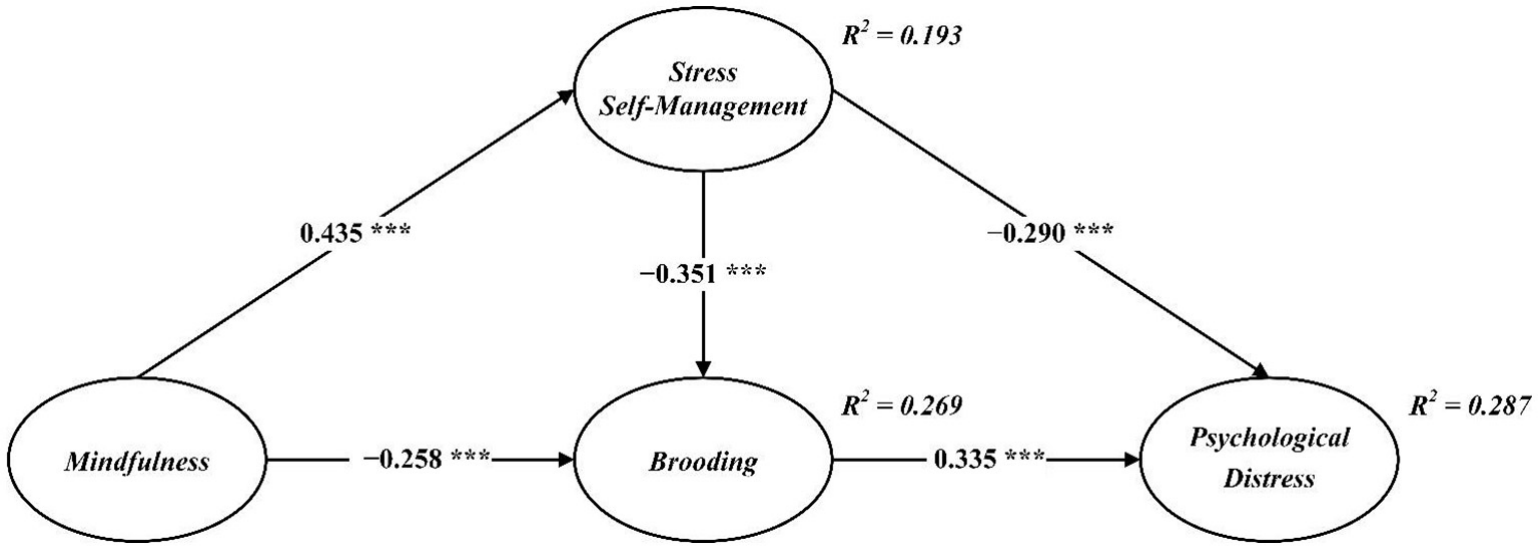
Structural model. Standardized coefficients are presented. Training frequency and sports injury experience were included as control variables in the analysis, but their paths are omitted from the figure for visual clarity. ***p < 0.001.

As shown in Figure 2, after controlling for training frequency and sports injury experience, mindfulness was positively associated with stress self-management (β = 0.435, *p* < 0.001), supporting H1. Mindfulness was negatively associated with brooding (β = −0.258, *p* < 0.001), supporting H2. Stress self-management was negatively associated with brooding (β = −0.351, *p* < 0.001), supporting H3. In addition, stress self-management was negatively associated with psychological distress (β = −0.290, *p* < 0.001), supporting H4, whereas brooding was positively associated with psychological distress (β = 0.335, *p* < 0.001), supporting H5.

Neither training frequency nor sports injury experience was significantly associated with the endogenous variables. Specifically, training frequency was not significantly associated with stress self-management (β = 0.003, *p* = 0.931), brooding (β = 0.016, *p* = 0.751), or psychological distress (β = 0.021, *p* = 0.675). Similarly, sports injury experience was not significantly associated with stress self-management (β = −0.063, *p* = 0.234), brooding (β = 0.018, *p* = 0.728), or psychological distress (β = 0.008, *p* = 0.871). Importantly, the inclusion of these control variables produced only negligible changes in the magnitude of the hypothesized relationships and did not alter their direction or statistical significance. Thus, the main findings remained robust after accounting for participants' training frequency and recent sports injury experience.

The explanatory power of the structural model was meaningful but moderate. Specifically, mindfulness, training frequency, and sports injury experience jointly explained 19.3% of the variance in stress self-management (*R*^2^ = 0.193). Mindfulness, stress self-management, training frequency, and sports injury experience jointly explained 26.9% of the variance in brooding (*R*^2^ = 0.269). Furthermore, stress self-management, brooding, training frequency, and sports injury experience jointly explained 28.7% of the variance in psychological distress (*R*^2^ = 0.287). Although these *R*^2^ values indicate that the proposed model accounted for a meaningful proportion of variance in the endogenous variables, they also suggest that a substantial proportion of variance remained unexplained. Therefore, other psychological, social, training-related, and contextual factors may also contribute to stress self-management, brooding, and psychological distress among taekwondo athletes.

### 4.3 Mediation analysis

Bootstrapping analysis was conducted to examine the indirect effect of mindfulness on psychological distress through stress self-management and brooding. As shown in Table 4, the standardized indirect effect was significant (β = −0.264, SE = 0.040, 95% CI [−0.342, −0.187], *p* < 0.001). Because the bias-corrected 95% confidence interval did not include zero, the mediating roles of stress self-management and brooding were supported. Therefore, H6 was supported.

**Table 4:** Standardized indirect effect.

Path	Point estimate	Product of coefficients	Bootstrapping		
			Bias-corrected 95% CI		Two-tailed significance
		SE	Lower	Upper	
MI → PD	−0.264	0.040	−0.342	−0.187	*p* < 0.001

## 5 Discussion

### 5.1 Theoretical contributions

The present study contributes to the existing literature on mindfulness and athletes' mental health in several important ways.

First, this study extends prior research on mindfulness and psychological functioning in sport settings by focusing specifically on taekwondo athletes, a population that has received comparatively limited scholarly attention. Previous studies have largely examined mindfulness and mental health among general athletic populations (Cui et al., 2025; Guendelman et al., 2017), while relatively fewer studies have focused on combat sports characterized by intensive physical confrontation, strict weight-management demands, and elevated injury risk. The present findings may be interpreted in light of these sport-specific demands. Taekwondo athletes often need to manage not only general competitive pressure but also repeated physical contact, rapid decision-making, weight-control stress, and injury concerns. In this context, mindfulness may be relevant because it is associated with present-moment awareness and reduced automatic engagement with negative thoughts, which may help athletes respond to pressure in a more regulated manner. By examining taekwondo athletes, the present study broadens the applicability of prior findings concerning mindfulness and psychological distress within a sport context involving distinctive psychological and competitive pressures.

At the same time, the findings should not be interpreted as showing that mindfulness is universally or equally protective across all athletic contexts. Prior research has not always produced entirely consistent results, with some studies suggesting that the association between mindfulness and mental health may vary depending on sport type, competitive level, training stage, intervention format, and athlete characteristics. Therefore, the contribution of the present study lies not in claiming a universal effect of mindfulness, but in showing that mindfulness is meaningfully associated with psychological distress among Chinese taekwondo athletes through specific self-regulatory and cognitive processes.

Second, this study contributes to the sport psychology literature by integrating both self-regulatory and cognitive processes into the same analytical framework. Previous research has demonstrated that self-regulatory capacities are important for athletes' psychological adaptation and coping under competitive conditions (Latinjak, 2025; García et al., 2023), while other studies have highlighted the role of maladaptive cognitive processes such as rumination and repetitive negative thinking in psychological maladjustment (Blankstein and Lumley, 2008; Maltais et al., 2019). However, these two perspectives have often been examined separately in the literature. The present findings suggest a possible explanatory process: athletes with stronger stress self-management may be better able to shift from emotional arousal to active coping, which may reduce the likelihood of becoming trapped in repetitive negative thinking. Conversely, when athletes engage in brooding, performance mistakes, injury concerns, or competitive setbacks may be repeatedly reprocessed in a negative way, thereby increasing vulnerability to psychological distress. The present study extends existing research by simultaneously examining stress self-management and brooding as interrelated psychological processes associated with mindfulness and psychological distress. The findings suggest that athletes' psychological distress may be understood not only in terms of adaptive behavioral regulation but also maladaptive cognitive regulation, thereby providing a more comprehensive understanding of athletes' mental health in competitive sport settings.

Third, the present study contributes to the application of self-regulation theory within sport psychology research. Self-regulation theory emphasizes that individuals continuously regulate their thoughts, emotions, and behaviors in response to environmental demands in order to maintain adaptive functioning (Balk and Englert, 2020). Although previous research has applied self-regulation theory to explain stress management, emotional regulation, and psychological adaptation in sport settings (Bakker and de Vries, 2021; Young et al., 2023), relatively limited research has simultaneously incorporated both adaptive self-regulatory behaviors and maladaptive cognitive regulatory tendencies when examining psychological distress among athletes. By conceptualizing stress self-management as an adaptive self-regulatory process and brooding as a maladaptive cognitive regulatory process, the present study extends the application of self-regulation theory to understanding psychological distress among taekwondo athletes. However, the moderate explanatory power of the model also indicates that self-regulatory and cognitive processes provide only a partial explanation of psychological distress. Other factors, such as training load, injury severity, social support, coach-athlete relationships, and recovery conditions, may also play important roles.

In summary, the present study enriches the literature on mindfulness and athletes' mental health by extending existing research to the context of taekwondo athletes and by integrating self-regulatory and cognitive processes within a unified analytical framework. By drawing upon self-regulation theory, this study provides a more comprehensive perspective for understanding how mindfulness is associated with psychological distress in high-pressure sport environments. Importantly, these findings should be interpreted as evidence of associations rather than causal effects, given the cross-sectional design of the study. Accordingly, the present study not only broadens current understanding of psychological distress among taekwondo athletes but also offers additional theoretical insight into the psychological processes underlying athletes' mental health and wellbeing.

### 5.2 Practical implications

The present findings provide several practical implications for different stakeholders involved in taekwondo training and athlete development. Given that mindfulness was associated with lower psychological distress both directly and indirectly through stress self-management and brooding, interventions targeting these psychological processes may be beneficial in athletic settings. However, these implications should be interpreted cautiously. The present study does not demonstrate that mindfulness training will necessarily reduce psychological distress, but it suggests that mindfulness, stress self-management, and brooding may be useful targets for future intervention and support programs.

First, for taekwondo athletes, the findings suggest that developing mindfulness may help reduce psychological distress by strengthening adaptive self-regulation and weakening maladaptive repetitive thinking. Athletes are often exposed to high training loads, competitive pressure, and injury-related stress. Under such conditions, improving present-moment awareness may help them better manage stressful experiences, regulate their daily routines, and reduce the tendency to dwell on negative thoughts. Therefore, athletes may benefit from regular mindfulness-based practices, such as breathing exercises, brief attention training, and moment-to-moment awareness practice integrated into daily training and recovery routines. These practices should be viewed as supplementary psychological skills rather than as substitutes for professional mental health support when athletes experience serious or persistent distress.

Second, for coaches, the results highlight the importance of paying attention not only to athletes' physical performance but also to their psychological coping patterns. Since stress self-management was negatively associated with both brooding and psychological distress, coaches may help athletes build healthier self-management habits, including maintaining adequate sleep, scheduling recovery time, and using simple stress-control strategies before and after training or competition. At the same time, coaches should be aware that athletes who repeatedly focus on mistakes, setbacks, or self-blame may be at greater risk of psychological distress. Rather than interpreting these signs as weakness or lack of motivation, coaches may encourage constructive reflection, provide supportive feedback, and refer athletes to qualified professionals when needed.

Third, for sports teams, clubs, and training institutions, the findings suggest that psychological wellbeing should be incorporated more systematically into athlete support systems. Rather than responding only after serious distress has emerged, organizations may consider implementing preventive mental health programs that include mindfulness training, stress management education, and early identification of maladaptive cognitive responses. Short workshops, routine psychological check-ins, and collaboration with sport psychologists may be useful ways to strengthen athletes' coping resources in a more sustainable manner. Such programs should be evaluated through longitudinal or intervention-based research before strong claims are made about their effectiveness.

Finally, for universities, sports schools, and policy makers in athlete development contexts, the findings indicate that athlete mental health should be addressed as part of holistic development rather than as a secondary issue. Educational and training systems may benefit from incorporating psychological skill development into regular athlete education programs. In particular, mindfulness and stress self-management skills may be embedded into athlete support curricula, while targeted guidance may be provided for those who are more vulnerable to brooding and distress. Such efforts may help promote not only psychological wellbeing but also more stable long-term athletic development. However, because the present study used a convenience sample of Chinese taekwondo athletes, future programs should be adapted and tested across different regions, competitive levels, and sport contexts before broader implementation.

### 5.3 Limitations

First, this study adopted a cross-sectional design, which limits the ability to draw causal conclusions. Although the structural model supported the hypothesized associations among mindfulness, stress self-management, brooding, and psychological distress, the direction of these relationships cannot be confirmed definitively. Future studies should employ more specific longitudinal designs, such as tracking taekwondo athletes across an entire competitive season. For example, researchers could collect data at multiple time points, including pre-season training, mid-season competition, major competition periods, and post-season recovery, to examine how mindfulness, stress self-management, brooding, and psychological distress change over time. Such a design would help clarify the temporal order and potential causal mechanisms among these variables. Future studies may also employ experimental designs, such as mindfulness-based or stress-management interventions, to further examine the causal mechanisms among these variables.

Second, the data were collected through self-report questionnaires from taekwondo athletes who were recruited using a convenience sampling approach. This may increase the risk of common method bias and limit the generalizability of the findings to other athlete populations or training contexts. Although Harman's single-factor test and the common latent factor approach suggested that common method bias was unlikely to be a serious concern, the study did not include a theoretically unrelated marker variable; therefore, the marker variable technique could not be applied. Future research could include marker variables, multi-source data, coach evaluations, psychological screening records, training-load information, injury records, or behavioral indicators of stress management to reduce common method bias and improve methodological rigor.

Third, the explanatory power of the proposed model was meaningful but moderate. The *R*^2^ values indicated that mindfulness, stress self-management, and brooding explained part of the variance in the endogenous variables, but a substantial proportion of variance remained unexplained. Therefore, the present model should be interpreted as a partial explanatory framework rather than a comprehensive account of psychological distress among taekwondo athletes. Future studies should include additional psychological, social, training-related, and contextual variables, such as training load, injury severity, competition level, coach support, social support, sleep quality, recovery status, and personality characteristics, to improve the explanatory power of the model.

## 6 Conclusion

This study examined the association between mindfulness and psychological distress among taekwondo athletes, with particular attention to the mediating roles of stress self-management and brooding. Drawing upon self-regulation theory, the findings indicated that mindfulness was positively associated with stress self-management and negatively associated with brooding, while stress self-management and brooding were further associated with psychological distress. In addition, mindfulness was indirectly associated with psychological distress through the mediating roles of stress self-management and brooding. These findings suggest that both adaptive self-regulatory processes and maladaptive cognitive processes are important for understanding athletes' psychological functioning in high-pressure sport environments. By focusing specifically on taekwondo athletes, this study extends the literature on mindfulness and athletes' mental health within a combat sport context characterized by intensive physical and psychological demands. Overall, the present study provides additional insight into the psychological processes associated with psychological distress among athletes and highlights the potential importance of mindfulness, stress self-management, and maladaptive cognitive tendencies in promoting athletes' mental health and wellbeing.

## Data Availability

The raw data supporting the conclusions of this article will be made available by the authors, without undue reservation.
